# P-22 - DO OUTBREAKS OF SEVERE PNEUMOCOCCAL DISEASE GO UNNOTICED IN CARE HOMES? A CASE SERIES FROM THE NORTH EAST OF ENGLAND

**DOI:** 10.1093/pubmed/fdag046.081

**Published:** 2026-07-09

**Authors:** Dr Gayle Dolan, Mr Jonathan Lawler, Mr Joe Jasperse

**Affiliations:** North East Health Protection Team, UK Health Security Agency; North East Health Protection Team, UK Health Security Agency; North East & Yorkshire and Humber Field Service, UK Health Security Agency

## Abstract

**Background:**

The Chief Medical Officer's Annual Report highlights the relative lack of attention that infection in older age receives. Respiratory infections are the most frequent cause of infection related deaths with pneumococcal disease a primary contributor, particularly amongst elderly care home residents. National guidance advises chemoprophylaxis and/or vaccination in the event of an outbreak and invasive Streptococcus pneumoniae (IPD) is a statutorily notifiable organism. In the North East, single cases of IPD and clusters of care home residents hospitalised with pneumonia are actively investigated although national gudiance advises that active surveillance is not an expectation of health protection teams (HPTs).

**Objectives:**

We describe a series of clusters of severe pneumococcal disease in elderly care home settings, including cluster identification, management and associated mortality and morbidity.

**Methods:**

Clusters actively managed by the North East HPT over a 15-month period (December 2024 to February 2026) were identified from the case management system CIMS. Data about cluster detection (including methods of reporting and microbiological investigation) and associated morbidity (hospitalisations and deaths) were extracted and described.

**Results:**

Six clusters (including a total of 18 confirmed cases) were identified and actively managed. None of the outbreaks had more than one case with a positive blood culture, relying on urinary antigen testing for detection of other cases. A total of 31 residents were hospitalised across all six outbreaks and there was at least one resident death in each situation. All six clusters required antibiotic prophylaxis, and vaccination was advised in five.

**Conclusions:**

Active investigation by the HPT identified clusters where public health action was indicated according to current guidance. These would have remained undetected if there was reliance on notification of positive blood culture results alone. Further evaluation of cluster detection methods including the role of urinary antigen testing should be explored.

